# Modeling the spread of the Zika virus using topological data analysis

**DOI:** 10.1371/journal.pone.0192120

**Published:** 2018-02-13

**Authors:** Derek Lo, Briton Park

**Affiliations:** 1 Department of Statistics, Yale University, New Haven, Connecticut, United States of America; 2 Department of Computer Science, Yale University, New Haven, Connecticut, United States of America; 3 Department of Mathematics, Yale University, New Haven, Connecticut, United States of America; Metabiota, UNITED STATES

## Abstract

Zika virus (ZIKV), a disease spread primarily through the *Aedes aegypti* mosquito, was identified in Brazil in 2015 and was declared a global health emergency by the World Health Organization (WHO). Epidemiologists often use common state-level attributes such as population density and temperature to determine the spread of disease. By applying techniques from topological data analysis, we believe that epidemiologists will be able to better predict how ZIKV will spread. We use the Vietoris-Rips filtration on high-density mosquito locations in Brazil to create simplicial complexes, from which we extract homology group generators. Previously epidemiologists have not relied on topological data analysis to model disease spread. Evaluating our model on ZIKV case data in the states of Brazil demonstrates the value of these techniques for the improved assessment of vector-borne diseases.

## Introduction

An explosive outbreak of ZIKV began in Brazil in April 2015. The ZIKV outbreak has spread to many regions throughout South America due to the abundance of the *Aedes aegypti* mosquito species [1], which is the primary transmission vector for ZIKV [2]. Approximately 2.6 billion people live in regions suitable for the virus to spread [3], and as of November 2016, 57 countries have active local ZIKV transmission [4]. Researchers project that the number of ZIKV cases in Brazil will be at least double that of any other country [5]. We focus our analysis on Brazil because it was one of the first countries to see an outbreak of ZIKV and therefore the virus has had more time to develop. The immediate symptoms of ZIKV are mostly mild, but ZIKV has been associated with more serious conditions. ZIKV has been linked to increased cases of the Guillain-Barré Syndrome [6], which is a severe neurological disease that causes the immune system to attack the nervous system. Occurrences of microcephaly, a condition causing the brain to be underdeveloped, has increased in the children of infected pregnant women [7]. Predicting the spread of ZIKV is, therefore, a priority.

Researchers have made substantial progress in modeling ZIKV to better understand and prevent its spread. Various models have already been developed to study the spread of vector-borne diseases. For example, variants of the SIR model from classic epidemiology theory can be used for these purposes and often are applied to obtain estimates of the basic reproduction number, a metric to study how infectious a disease is [8]. For example, Gao *et al*. (2016) used a type of SEIR model based on classic epidemic theory to estimate the impact of mosquito-borne and sexual transmission of the ZIKV in Brazil, Colombia, and El Salvador and estimate a basic reproduction number of the virus [9]. However, this framework requires reliable estimates of epidemiological parameters such as the vector disease transmission rate.

Another class of models that are often used by researchers to study the spread of vector-borne diseases are time series models such as ARIMA [10]. These models can take advantage of time series data of climate conditions and disease incidence data to model disease transmission over time. Zhang *et al*. (2016) developed a time series predictive model for the dengue disease in China using weather predictions and dengue surveillance information in order to obtain projections of the number of ZIKV infections in the Americas [11]. One drawback of time series models is that acquiring granular time-series data over extended durations can be difficult [11].

Researchers have also used stochastic models to analyze ZIKV spread and prevention methods. Castro *et al*. (2016) developed a stochastic model to capture the uncertainty in ZIKV reporting, importation, and transmission to identify regions of greatest risk in the state of Texas [12]. But again, this model again requires estimates of epidemiological parameters like reporting rates and vector abundance [12].

We propose studying the topological features of vector locations to inform the parameter estimation of these models. The primary benefit of using topological data analysis over the traditional models mentioned above is that it allows researchers to gain information from spatial data in a computationally efficient way. Spatial data is prevalent in many applications in studying disease spread, which makes topological data analysis an especially exciting new tool for epidemiologists. To validate the explanatory power of these features in this specific application, we use a linear regression model to predict the number of ZIKV cases in each state of Brazil using features generated from the mosquito population topology. We find that our prediction results are comparable to the performance of models in the existing literature.

## Methods

Predictions of the number of Zika cases can be obtained by utilizing the population density of *Aedes aegypti* mosquitoes [13], average temperature of a given region [14], and human population density. However, by applying techniques from persistent homology via Vietoris-Rips filtrations—we find valuable information within the spatial structure of the locations of *Aedes aegypti* mosquitoes.

**Fig 1:**
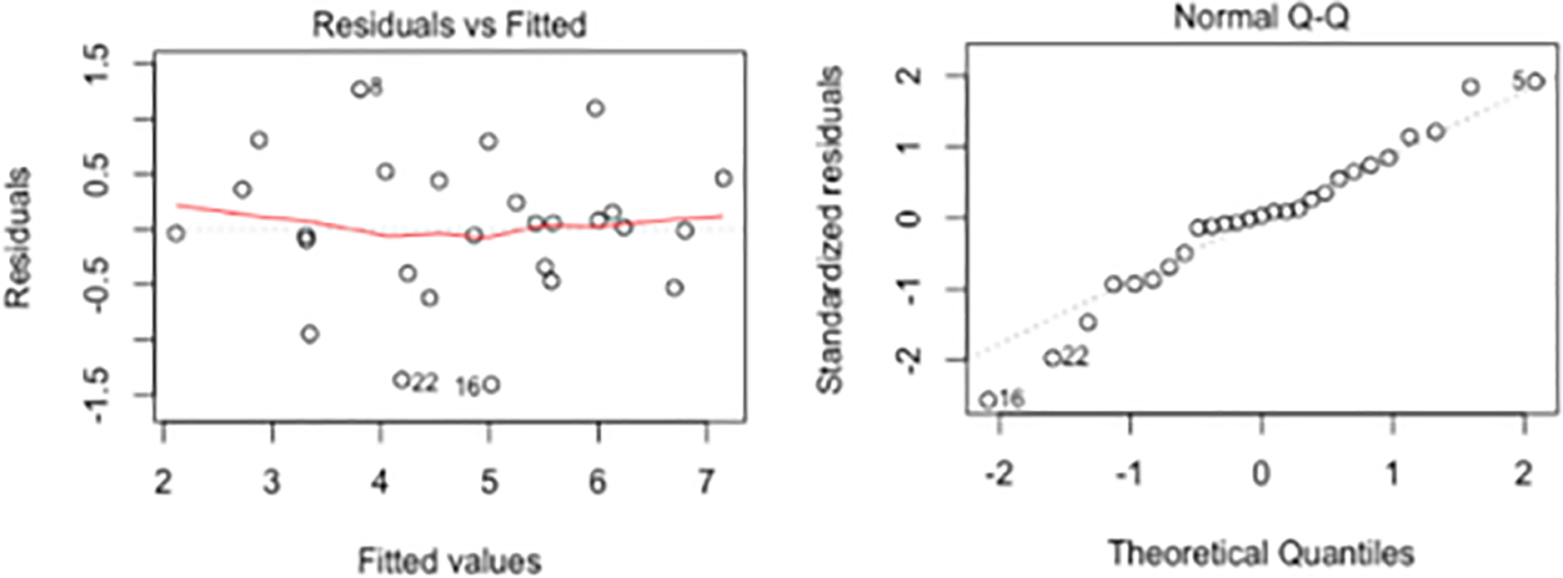
Fitted vs residuals plot and Q-Q plot of standardized residuals of model A. The two plots show that the residuals seem to be symmetrically distributed around 0 and in general clear patterns are not present. Therefore, the residuals are approximately normally distributed around 0 and a linear regression with a logarithmic transformation of the number of ZIKV cases appropriately models our data.

First, we obtain data on the geographic locations of *Aedes aegypti* mosquitos in Brazil. In 2013, Brazilian municipalities conducted physical household surveys, searching for mosquito larvae, pupae, and adult mosquitos. If a mosquito population was discovered in the municipality over a region greater than 5km x 5km, a “mosquito occurrence” was marked as a polygon region [15]. The coordinate associated with this polygon its centroid. There are 5057 entries, each of which has an associated polygon centroid that represents a mosquito population at that location during 2013 [16]. An example of our data for Brazil and the state of Sergipe is given in Fig 1. Also, it is well known that *Aedes aegypti* mosquitos thrive in warmer weather (up to 32° C) and in regions with higher rainfall [17]. Using this information, we weight municipalities that are more suitable to *Aedes aegypti* reproduction. To estimate the relationship between the number of ZIKV cases and the spatial structure of the *Aedes aegypti* mosquito populations within each state, we use monthly reports containing the cumulative number of confirmed ZIKV cases in each state, which Brazil's Ministry of Health has been publishing since 2015.

We propose a model that exploits the spatial information within the *Aedes aegypti* mosquito occurrence maps. This is done by utilizing ideas from persistent homology to extract topological information from the 2-dimensional point clouds resulting from the longitudinal and latitudinal coordinates of the *Aedes aegypti* mosquito polygon centroids. Specifically, we study the 0^th^ and 1^st^ dimensional homology group generators (connected components and loops), which cannot be accessed using more standard statistical techniques. We use the Vietoris-Rips filtration [18, 19] to create intermediate structures (simplicial complexes) from which we can extract topological information from our original data. The Vietoris-Rips filtration is applied to the coordinates of the polygon centroids in each state of Brazil using the TDA package in R [18]. The filtration is constructed starting with balls of radius 0 around each point. As we grow the ε-balls, some begin to intersect with one another, which forms “simplexes”. A 0-simplex is defined to be a single vertex, a 1-simplex is a line segment connecting a pair of vertices and a 2-simplex is a triangle connecting three vertices. For each value of ε, we obtain a simplicial complex which is composed of all the simplexes in the filtration. Note that in the filtration, the simplicial complex for a specified value of ε is a subset of the simplicial complexes of larger ε.

Using these topological features of the *Aedes aegypti* mosquito occurrence locations in each state of Brazil and the corresponding number of ZIKV cases in each state, we fit linear regression models. More specifically, we look at the number of H0 features at the start of the filtration (H0N), the total number of H1 features throughout the filtration (H1N), and the maximum lifetime of H1 features (H1ML). The H0N features measure the density of mosquitoes, with a higher number of H0 features indicating a greater presence of mosquitos. The H1 features measure the spatial distribution of mosquitoes, with more H1 features indicating loops or areas in the state without mosquito occurrences.

## Results and discussion

Researchers have found the effects of mosquito populations and climate to be useful in anticipating the transmission of vector-borne diseases [14, 20]. Some have even proposed a fast way of predicting the spread of ZIKV infections in Brazil [5, 13], which involves estimating the number of ZIKV cases using attributes such as the average temperature, the population density, and the population of *Aedes aegypti* mosquitos of a region. We use the number of *Aedes aegypti* mosquito occurrences (AMO) in the 2013 physical household surveys in Brazil described above as a proxy for the *Aedes aegypti* mosquito population for each state of Brazil. For the temperature of each state, we use the mean yearly temperature calculated by averaging monthly temperatures in degrees Celsius of 98 weather stations in Brazil in 2010 (TEMP) [21]. To estimate the population density, we divide the estimated resident population size of each state in 2014 by the geographic area of each state in km^2^ (HPOP) [22].

We propose a model that takes advantage of the spatial information within *Aedes aegypti* mosquito occurrences maps in addition to state-level attributes such as population density and average temperature. We include the number of H0 features to predict the number of ZIKV cases as a topological feature, since states with fewer H0 features have fewer municipalities that have had occurrences of the *Aedes aegypti* mosquito than states with less H0 features. A low number of H0 features for a state could either arise through a fewer number of municipalities or the existence of municipalities that do not harbor *Aedes aegypti* mosquitos. Because ZIKV is mainly transmitted to people from *Aedes aegypti* mosquitoes, either case will lead to fewer infections. A fewer number of municipalities is likely to indicate a lower population density in a state, which would decrease the chance of ZIKV infections, while the existence of municipalities that do not harbor many *Aedes aeypgti* mosquitoes also can lead to a lower chance of ZIKV infections for a state due to a fewer number of transmission vectors. Therefore, we anticipate that ZIKV transmission to people is more likely in the states with more occurrences of the *Aedes aegypti* mosquito or H0 features and less likely in states with fewer occurrences of the mosquito.

We also include the number of H1 features to model the spread of ZIKV. A large number of H1 features within a state may arise due to a large amount of municipalities with *Aedes aegypti* mosquitoes exist within a state, which create more loops during the Vietoris-Rips filtration due to chance or municipalities in general are more spread out due to geographic barriers such as mountains or lakes. Keeping the number of municipalities with *Aedes aegypti* mosquitoes constant within a state, we anticipate that more H1 features will signify that municipalities with the mosquito occurrences are spread out. This spatial distribution of these municipalities may indicate either a low *Aedes aegypti* mosquito density or human population density in a state. Therefore, we anticipate that a large number of H1 features will have a negative effect on the number of ZIKV infections, keeping the number of H0 features constant. It is worth noting that the H1 features respect geographical topology such as lakes and mountainous regions, since municipalities are far less likely to be located in these areas. Thus it is very improbable to have polygon centroids located in such regions and therefore the loops do not intersect a state’s topography.

Lastly, we include H1ML in our model because it informs the disease’s transmission rate between municipalities. States with densely packed municipalities will see higher transmission rates due to greater mobility. And conversely, states with municipalities spread far apart due to geographic topology such as mountainous regions and lakes will observe lower transmission rates, and therefore fewer cases. A high H1ML indicates that the municipalities within that state are spread farther apart than states with low H1ML. Furthermore, simply calculating the density of municipalities is not sufficient; the state could be very large and have a few number of closely packed municipalities. Thus, we need some way of determining the proximity of the municipalities between each other. The H1ML feature gives us a proxy for this. We specifically study the maximum lifetime of H1 features for each state rather than an average or median, since this metric is robust to the H1 features that have very short lifetimes which may arise due to a large number of H0 features out of chance rather than being a signal for areas free of *Aedes aegypti* mosquitos.

Using these features, we create a linear regression model. This model uses AMO (H0N), TEMP, POP, H1N, H1ML as its predictors and the number of ZIKV cases with a logarithmic transformation as the response. The model is labeled A (Table 1). All of the model’s features are statistically significant at the 5% level. AMO (H0N), POP, and TEMP have positive effects on the number of ZIKV cases, while the interaction between H1ML and H1N have negative effects. To compute the fit of the model, we calculate the model’s adjusted *R*^*2*^ and find it to be 0.76. Additionally, to test if a linear regression model is appropriate for the data, we look at the residuals of the model (Fig 1). We find that the residuals are approximately normally distributed, which imply that a linear regression model is appropriate. To test how well the model predictions perform, we use leave-*p*-out cross-validation and achieve average errors of 0.75, 0.40, and 0.79 using *p =* 1, 2, and 3, respectively.

**Table 1:** Coefficients of linear regression model predictors.

Model	Model A	Model B
Intercept	-3.03	-3.72
AMO / H0N	0.057*	0.0067*
H1N	-0.12*	-
Interaction (H1N and H1ML)	-0.090*	-
POP	0.0056*	0.0060*
TEMP	0.20*	0.27*

* Coefficient is statistically significant at the 5% significance level

It is possible that this final model overfits the data given its higher number of topological predictors. To check for this potential problem, we fit another linear regression model by removing the topological features predictors; this model is labeled B in Table 1. The model’s goodness-of-fit can be measured through the adjusted *R*^2^, which was 0.42. We also apply leave-*p*-out cross-validation [23] using *p =* 1, 2, and 3 to test how well the model predicts the log-transformed number of ZIKV cases in each state of Brazil. The model achieves an average cross-validated squared error of 1.89, 1.97, and 2.05 for *p* = 1, 2, and 3, respectively (Table 2). Therefore, we see that model A is an improvement over the reduced model B when evaluating them on the prediction errors, as well on the adjusted *R*^*2*^ metric.

**Table 2:** Leave-*p*-out cross-validation mean squared errors.

*p*	Model A	Model B
1	0.75	1.89
2	0.40	1.97
3	0.79	2.05

**Fig 2:**
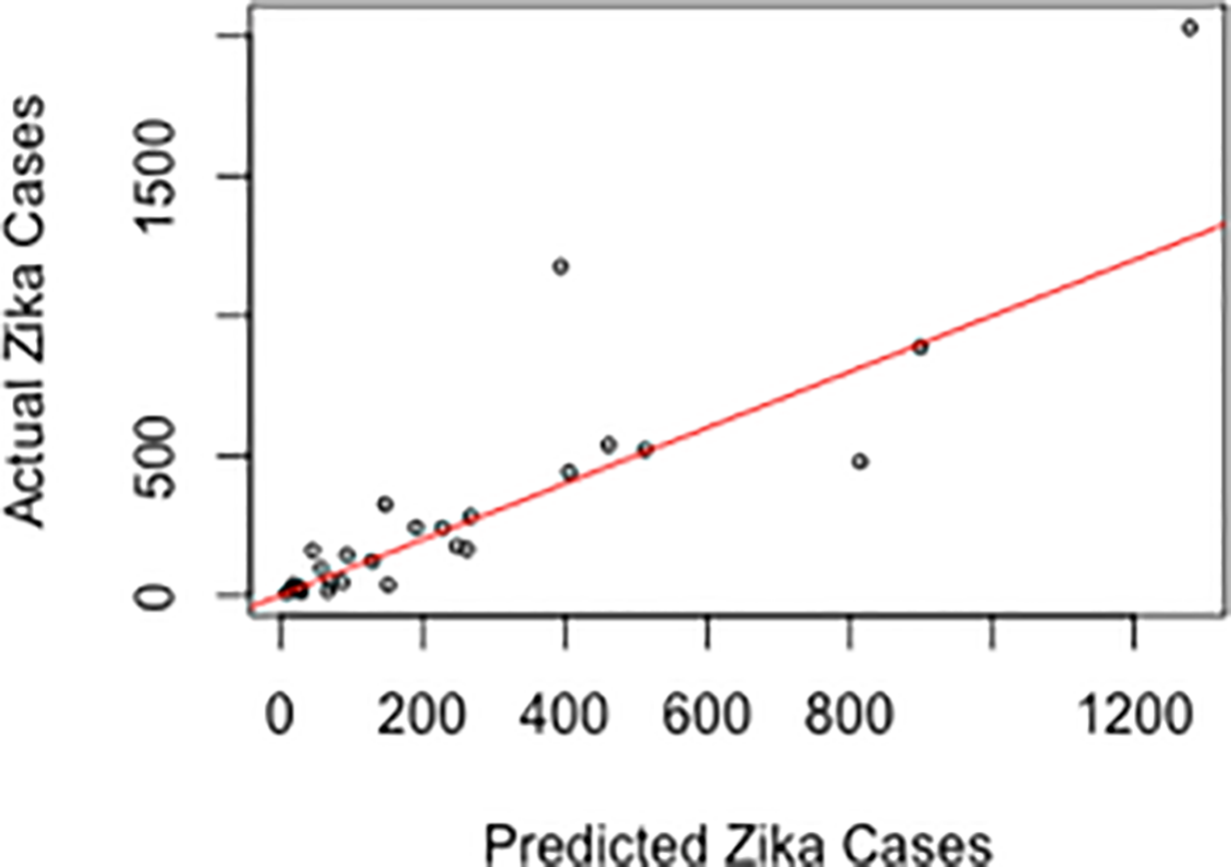
Predicted versus actual confirmed zika cases for July 2, 2016. We plot the predicted ZIKV cases against the confirmed Zika cases. The correlation between the predictions and the confirmed cases is 0.71.

We compare the results of our model with the results of Zhang *et al*. (2016). Zhang *et al*. (2016) report a Pearson correlation value of 0.57 between state-level model projections of ZIKV cases and surveillance data of ZIKV cases through June 2016 in Colombia. We obtain a Pearson correlation value of 0.88 between state-level model projections of ZIKV cases and the cumulative number of actual ZIKV cases through July 2016 in Brazil and plot the predicted number of cases against the confirmed ZIKV cases (Fig 2). Due to our high correlation value, our model performance is comparable to the results in Zhang *et al*. (2016). Thus, we show the explanatory power of the integration of state-level attributes and topological features in predicting the number of ZIKV cases through using even simple linear regression.

Overall, we have shown that topological features of the locations of mosquito occurrences contain additional information that can be used in conjunction with standard features to better predict the spread of ZIKV. Due to the nature of vector-borne diseases, infected arthropod species are their primary modes of transmission. Our results suggest that applying TDA to their locations can help epidemiologists and public-health officials better track vector-borne diseases and curb the spread of future contagions.

## Supporting information

S1 File This document contains a brief tutorial of topological data analysis and a more detailed description of the statistical methods we used in this paper. Finally, the supporting information also contains the code we used for our analysis and where we collected our data.
(PDF)
