# Supplementary material for: Modeling the spread of the Zika virus using topological data analysis
Source: PLoS One. 2018 Feb 13;13(2):e0192120. doi: 10.1371/journal.pone.0192120 (PMC5810985; doi:10.1371/journal.pone.0192120)
Supplement: S1 File — Finally, the supporting information also contains the code we used for our analysis and where we collected our data. (PDF) [file pone.0192120.s001.pdf]

Modeling the spread of the Zika virus using topological data analysis

Derek Lo[1,2,*,†] and Briton Park[1,3,†]

[1] Department of Statistics, Yale University, New Haven, CT
[2] Department of Computer Science, Yale University, New Haven, CT
[3] Department of Mathematics, Yale University, New Haven, CTs
[†] Authors are co-first authors listed in alphabetical order by last name
[*] Email: derektlo@gmail.com

Table of Contents

Methods

1. Topological Data Analysis
1.1 Persistent Homology
1.2 Introduction to the Vietoris-Rips Filtration
1.3 R code for Vietoris-Rips Filtration
1.4 Persistence Diagram

2. Statistical Analysis
2.1 Modeling using topological features
2.2 R code for modeling

3. Code and Data Availability
3.1 Aedes aegypti Mosquitos
3.2 Temperature
3.3 Population Density
3.4 Zika Cases

References

Methods

1. Topological Data Analysis

Topological data analysis (TDA) is a growing field of statistics that makes use of techniques from topology to analyze data. We believe TDA provides valuable tools that epidemiologists can use to improve modeling the spread of disease. Specifically, we will explain the very basics of persistent homology, such as the Vietoris-Rips filtration, as well as how to carry out the filtration using the R programming language. Finally, we explain the persistence diagram, which is a common way of visualizing and extracting information from the Vietoris-Rips filtration.

1.1 Persistent Homology

The central idea of persistent homology is to compute topological features of data through a series of polyhedra, which are simplicial complexes based on a parameter, ε. First to understand what a simplicial complex is, we define the notion of a simplex. In geometry, a simplex is a generalization of a triangle or tetrahedron to n dimensions. For example, a k-simplex is a k-dimensional convex hull composed of k + 1 vertices (a 2-simplex is a triangle). A simplicial complex is then a combination of simplices. Each simplicial complex is contained in the subsequent simplicial complex with a larger ε parameter. After obtaining a simplicial complex, its homology vector space can be computed. Each element of the homology vector space represents a type of structure in the complex. But does this structure also exist in the data? To answer this question, we track each homology element (or homology class) as the ε parameter grows. If the feature is statistical noise, the existence of that homology class will likely be short. If the feature is a real signal, the homology class is likely to persist for a longer time[1]. Hence, this method is called *persistent* homology.

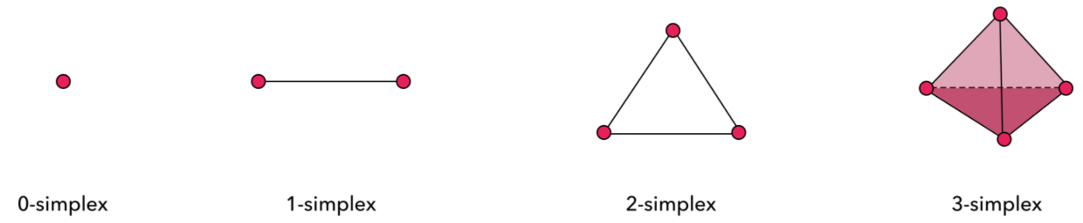

S1 Fig. Example simplex visualizations. An example of simplexes from dimension 0 to dimension 3. These are used to build a simplicial complex, which is the product of a Vietoris-Rips Filtration. See section 1.2 for a detailed walkthrough of this filtration.

1.2 Introduction to the Vietoris-Rips Filtration

The Vietoris-Rips filtration is a popular tool in topological data analysis because it can be used to encode valuable information about the underlying topology of data. The filtration produces a set of simplicial complexes that add a topological skeleton to point clouds. However, it is good to

note that in practice, datasets are often too large for the filtration to be calculated in a computationally feasible manner.

To build a complex from a point cloud data set using the Vietoris Rips filtration, we want to approximate the data by a polyhedron, which is a simplicial complex. We define the simplex inductively. The 0-simplices, the vertices of the complex, are defined by the data points. We first initialize a small parameter ε starting at an ε of 0. We surround each vertex with a 2D ball with radius equal to ε and grow these ε balls. If two ε balls intersect, we create a 1-simplex by joining the two 0-simplices at the center of these balls to create a line segment. If 3 ε balls intersect, we create a 2-simplex by joining the three 0-simplices at the center of the 3 balls in a triangle. Now we construct the homology vector spaces by finding the cycles in the complex which are the generators of the homology vector space. A cycle is a simplex which is *not* part of the boundary of a simplex of higher dimension. For example, the vertices of a 1-simplex are not cycles. The components of the polyhedron are the 0-cycles. A loop of 1-simplices, which is not the boundary of a 2-simplex (triangles in the complex) is a 1-cycle. So the 0-cycles count the components of the polyhedron and the 1-cycles count the non-trivial loops in the polyhedron. Basically the cycles are just the holes of various dimension in the complex. These cycles are the generators of the homology vector spaces denoted by H0, H1, H2 for each dimension. The dimension of the vector spaces H0N, H1N are the indices we compute and interpret for the mosquito data.

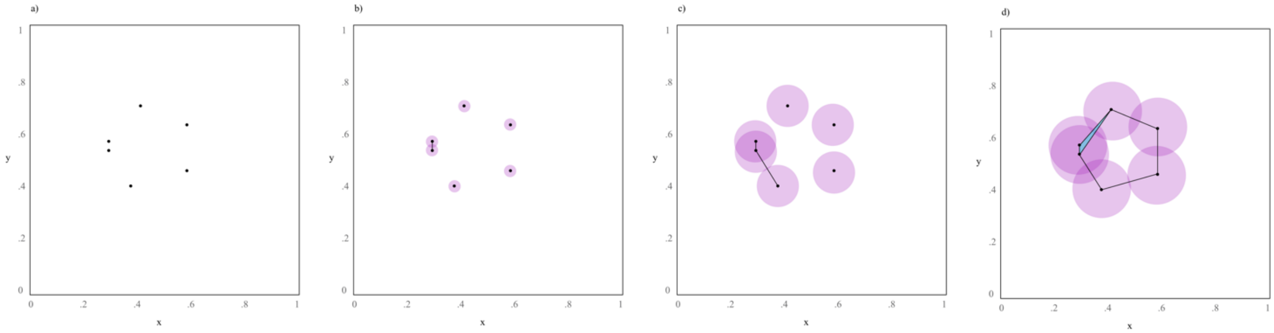

S2 Fig. Vietoris-Rips at increasing values of ε. A visualization at various ε values of the Vietoris-Rips filtration applied to a toy dataset. Notice how in panel a, we begin with ε = 0 since there are no balls present, and we have six 0-simplices. In panel b we begin to grow ε, and we observe that two points become connected to each other, forming our first 1-simplex. In panel c as we grow ε even further, another pair of points becomes connected, birthing another 1-simplex. Finally, in panel d ε has reached a large enough value that an open loop forms, as well as a closed loop in blue.

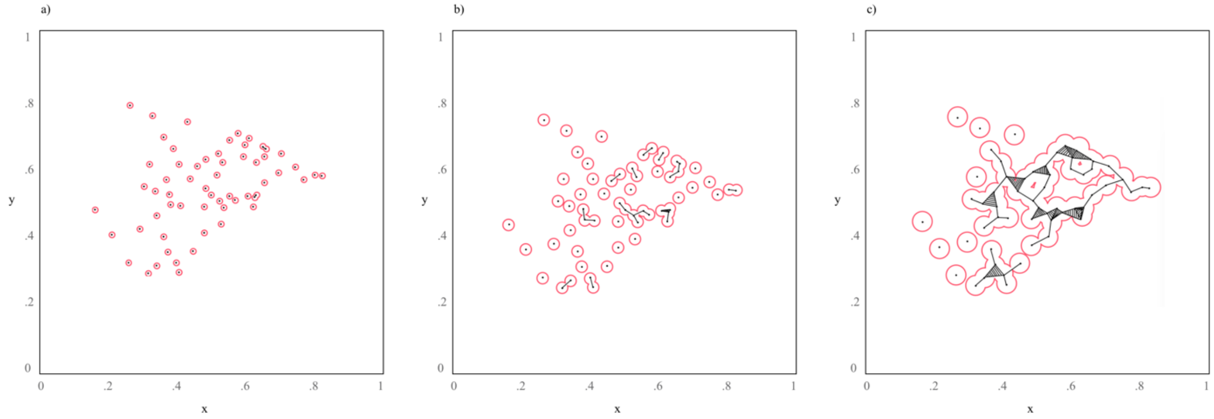

S3 Fig. Vietoris-Rips snapshots for Sergipe Aedes aegypti mosquito locations. Snapshots of the Vietoris-Rips filtration applied to Aedes aegypti mosquito population polygon centroid locations in Sergipe at several ε values. a-c**,** Depictions of the Vietoris-Rips filtration on the Sergipe mosquito map for $\varepsilon = 0.1$, 0.2, and 0.5. Black dots represent 0-simplexes, lines represent 1-simplexes, and shaded triangles represent 2-simplexes, while red circles denote ε-balls. 2-simplexes are filled because they are, by definition, closed loops.

There are several existing software packages that can compute the Vietoris-Rips filtration, including the TDA package in R, Perseus, and Dionysus.

1.3 R code for Vietoris-Rips Filtration

```
library(TDA)

Sao_Paulo <- ripsDiag(states[states$V13 == "Sao Paulo",c(6,7)], 1, 3, dist = "euclidean")
plot(Sao_Paulo$diagram, main = "Sao_Paulo, 479", band = 2*cc1_0)

Maranhao <- ripsDiag(states[states$V13 == "Maranhao",c(6,7)], 1, 2, dist = "euclidean")
plot(Maranhao$diagram, main = "Maranhao, 282")

Santa_Catarina <- ripsDiag(states[states$V13 == "Santa Catarina",c(6,7)], 1, 20, dist =
"euclidean")
plot(Santa_Catarina$diagram, main = "Santa Catarina, 8")

Amapa <- ripsDiag(states[states$V13 == "Amap\xcc\xc1",c(6,7)], 1, 20, dist = "euclidean")
plot(Amapa$diagram, main = "Amapa, 11")

Acre <- ripsDiag(states[states$V13 == "Acre",c(6,7)], 1, 5, dist = "euclidean")
plot(Acre$diagram, main = "Acre, 40")

Alagoas <- ripsDiag(states[states$V13 == "Alagoas",c(6,7)], 1, 0.8, dist = "euclidean")
plot(Alagoas$diagram, main = "Mato Alagoas, 325")

Amazonas <- ripsDiag(states[states$V13 == "Amazonas" | states$V13 == "State of Amazonas",c(6,7)],
1, 10, dist = "euclidean")
plot(Amazonas$diagram, main = "Amazonas, 25")

Bahia <- ripsDiag(states[states$V13 == "Bahia",c(6,7)], 1, 2, dist = "euclidean")
plot(Bahia$diagram, main = "Bahia, 1175")
```

```
Ceara <- ripsDiag(states[states$V13 == "Cear\xcc\xc1",c(6,7)], 1, 1, dist = "euclidean")
plot(Ceara$diagram, main = "Ceara, 521")

Espirito_Santo <- ripsDiag(states[states$V13 == "Esp\xcc_rito Santo",c(6,7)], 1, 1, dist =
"euclidean")
plot(Espirito_Santo$diagram, main = "Espirito_Santo, 160")

Gois <- ripsDiag(states[states$V13 == "Goi\xcc\xc1s",c(6,7)], 1, 1.5, dist = "euclidean")
plot(Gois$diagram, main = "Gois, 145")

Mato_Grosso <- ripsDiag(states[states$V13 == "Mato Grosso",c(6,7)], 1, 2, dist = "euclidean")
plot(Mato_Grosso$diagram, main = "240")

Mato_Grosso_Sul <- ripsDiag(states[states$V13 == "Mato Grosso do Sul",c(6,7)], 1, 3, dist =
"euclidean")
plot(Mato_Grosso_Sul$diagram, main = "Mato Grosso Sul, 22")

Minas_Gerais <- ripsDiag(states[states$V13 == "Minas Gerais",c(6,7)], 1, 2, dist = "euclidean")
plot(Minas_Gerais$diagram, main = "Minas Gerais, 122")

Para <- ripsDiag(states[states$V13 == "Par\xcc\xc1",c(6,7)], 1, 3, dist = "euclidean")
plot(Para$diagram, main = "Para, 46")

Paraiba <- ripsDiag(states[states$V13 == "Para\xcc_ba",c(6,7)], 1, 0.6, dist = "euclidean")
plot(Paraiba$diagram, main = "Paraiba, 889")

Parana <- ripsDiag(states[states$V13 == "Paran\xcc\xc1",c(6,7)], 1, 1.5, dist = "euclidean")
plot(Parana$diagram, main = "Parana, 37")

Pernam <- states[states$V13 == "Pernambuco",c(6,7)]
Pernam <- Pernam[-(nrow(Pernam)-1),]
Pernambuco <- ripsDiag(Pernam, 1, 6, dist = "euclidean")
plot(Pernambuco$diagram, main = "Pernambuco, 2029")

Piaui <- ripsDiag(states[states$V13 == "Piau\xcc_",c(6,7)], 1, 2, dist = "euclidean")
plot(Piaui$diagram, main = "Piaui, 176")

Rio_de_Janeiro <- ripsDiag(states[states$V13 == "Rio de Janeiro",c(6,7)], 1,1, dist =
"euclidean")
plot(Rio_de_Janeiro$diagram, main = "Rio de Janeiro, 537")

Rio_Grande_do_Norte <- ripsDiag(states[states$V13 == "Rio Grande do Norte",c(6,7)], 1, 1.2, dist
= "euclidean")
plot(Rio_Grande_do_Norte$diagram, main = "440")

Rio_Grande_do_Sul <- ripsDiag(states[states$V13 == "Rio Grande do Sul",c(6,7)], 1, 3, dist =
"euclidean")
plot(Rio_Grande_do_Sul$diagram, main = "Rio Grande do Sul, 96")

Rondnia <- ripsDiag(states[states$V13 == "Rondnia",c(6,7)],  2, 2, dist = "euclidean")
plot(Rondnia$diagram, main = "Rondnia, 17")

Roraima <- ripsDiag(states[states$V13 == "Roraima",c(6,7)], 1, 2, dist = "euclidean")
plot(Roraima$diagram, main = "Roraima, 26")

Sergipe <- ripsDiag(states[states$V13 == "Sergipe",c(6,7)], 1, 1, dist = "euclidean")
plot(Sergipe$diagram, main = "Sergipe, 242")

Tocantins <- ripsDiag(states[states$V13 == "Tocantins",c(6,7)], 1, 1.5, dist = "euclidean")
```

```
plot(Tocantins$diagram, main = "Tocantins, 164")
```

1.4 Persistence Diagram

Typically, persistent homology is visualized through either a barcode diagram or a persistence diagram. A persistence diagram is a plot of the birth and death times of each topological feature in every dimension. The first coordinate of each point denotes the birth time of the feature, while the second coordinate denotes the death time. Formally, a persistence diagram is a set of points expressed as: $\{(u,v) \in R^2 \mid u,v \geq 0, u \leq v\}$. Therefore, all points lie on or above the line of equality. Points close to the line of equality denote features with shorter lifetimes, while points that lie farther away from the line denote features that persist for a longer time.

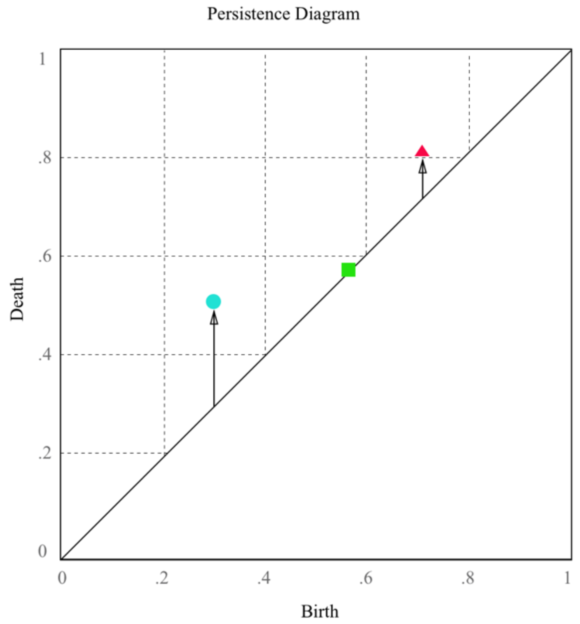

S4 Fig. Example persistence diagram. A persistence diagram that summarizes information from a Vietoris-Rips filtration. The lengths of the vertical arrows represent the lifetime of the respective features that they point to. The green square is a feature that was born and died at the same time. This can happen when a loop is formed but is immediately closed at once.

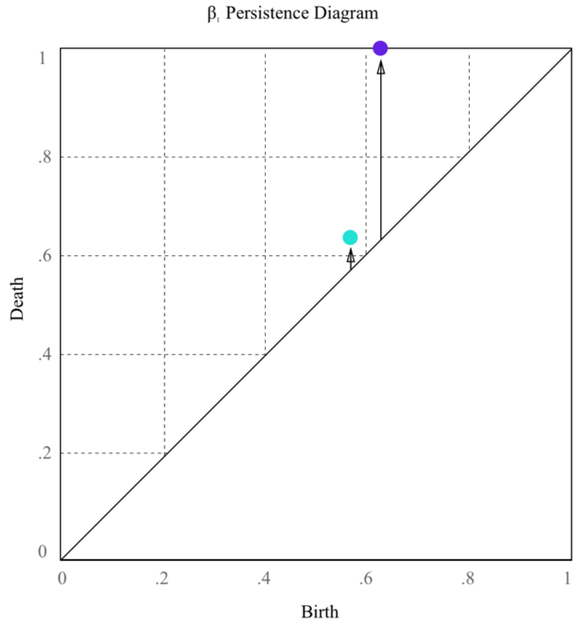

S5 Fig. Persistence diagram of 1D features. Persistence diagram that depicts specifically the 1D topological features of the example filtration from 1.2, visualized in S1 Fig. Notice that the blue circle has a very short lifetime, and it corresponds to the blue closed triangle in panel d of S1 Fig. In that same panel, we notice that a large outer loop has formed with all of the points. This corresponds to the purple point, which has a death time of 1 since the feature lives past the end of the filtration.

2. Statistical Analysis

2.1 Modeling using topological features

We are interested in studying the 0-dimensional homology group generators (H0 features) and 1-dimensional homology group generators (H1 features) of the simplicial complexes representing the original point clouds of mosquito occurrences of each state of Brazil. The number of H0 features at the the beginning of the filtration correspond to the number of *Aedes aegypti* mosquito occurrences in each state, since each occurrence starts out as a separate connected component as 0-simplexes in the filtration. Therefore, the H0 features all have birth time of $\varepsilon = 0$. As we grow ε, 1-simplexes are created from intersections of the ε-balls, and the number of connected components decreases. When a connected component dies by joining another connected component, we note its death time or the ε value at which the component dies. The H1 features correspond to the empty loops born in the Vietoris-Rips filtration. The H1 features die in the filtration when they are filled in by 2-simplexes. We note the birth and death time of these topological features, which are visually displayed in persistence diagrams. We then compute the lifetimes of the H1 features using the birth and death times in the persistent diagrams and record the maximum lifetime for the H1 features for each filtration.

2.2 R Code for Modeling
I. Linear Regression

```
lmA <- lm(log(Brazil$Cases_latest) ~   Brazil$Lifetime_1D*Brazil$Number_1D + Brazil$Number_0D
+Brazil$temp + Brazil$Population_density )

lmB <- lm(log(Brazil$Cases_latest) ~ Brazil$Number_0D +  Brazil$Population_density + Brazil$temp)

summary(lmA)
summary(lmB)
AIC(lmA)
BIC(lmB)
```

II. Cross Validation

```
### Leave-1-out cross validation

error12 <- rep(0,27)
error1 <- rep(0,27)

Brazil$pred1 <- 0
Brazil$pred12 <- 0
for(i in 1:27){
  A <- Brazil[i,]
  B <- Brazil[-i,]
  lm1 <- lm(log(Cases_latest) ~ Number_0D +  Population_density + temp, data = B)
  lm12 <- lm(log(Cases_latest) ~ Lifetime_0D_R*Number_1D + Number_0D  + temp  +
Population_density, data = B)
  Brazil$pred1[i] <-  exp(predict(lm1, A))
  Brazil$pred12[i] <-  exp(predict(lm12, A))
  error1[i] <-  log(A$Cases_latest) - predict(lm1, A)
  error12[i] <- log(A$Cases_latest) - predict(lm12, A)
}

sum(error1**2)/27
sum(error12**2)/27

### Leave-2-out cross validation

error1 <- rep(0,351)
error12 <- rep(0,351)
count <- 0
for(i in 1:26){
  for(j in (i+1):27){
      count <- count + 1

      train <- Brazil[-c(i,j),]
      test <- Brazil[c(i,j),]
      lm1 <- lm(log(Cases_latest) ~ Number_0D +  Population_density + temp, data = train)
      lm12 <- lm(log(Cases_latest) ~Number_1D + Life_time_0D_R + Lifetime_1D*Number_0D + temp +
Population_density, data = B)
      error1[count] <-  mean((log(test$Cases_latest) - predict(lm1, test))**2)
      error12[count] <- mean((log(test$Cases_latest) - predict(lm12, test))**2)
  }
}
sum(error1)/351
sum(error12)/351
```

```
### Leave-3-out cross validation
X <- combn(1:27, 3)
count <- 0
error1 <- rep(0,2925)
error12 <- rep(0,2925)
for(i in 1:2925){
  count <- count + 1
  train <- Brazil[-X[,i],]
  test <- Brazil[X[,i],]
  lm1 <- lm(log(Cases_latest) ~ Number_0D +  Population_density + temp, data = train)
  lm12 <- lm(log(Cases_latest) ~Number_1D + Life_time_0D_R + Lifetime_1D*Number_0D + temp +
Population_density, data = train)

  error1[count] <-  mean((log(test$Cases_latest) - predict(lm1, test))**2)
  error12[count] <- mean((log(test$Cases_latest) - predict(lm12, test))**2)
}
mean(error1)
mean(error12)
```

3. Code and Data Availability

All of the data used for analysis in this paper is 3rd-party data. We had no special privileges to access the data.

***Aedes aegypti* Mosquitos**

Our mosquito data comes from the Global Compendium of *Aedes aegypti* and *Ae. albopictus* occurrence dataset [2]. The full dataset is hosted by the Dryad Digital Repository at http://dx.doi.org/10.5061/dryad.47v3c. In total we consider 5057 entries for Brazil. Each entry represents a mosquito population in a region called a polygon, which is an area with dimension greater than 5km x 5km. Each polygon represents a survey conducted within a Brazilian municipality with a positive finding of a mosquito population of non-zero size. No attempt was made to quantify the true number of mosquitos within a polygon. There are 5570 municipalities in Brazil.

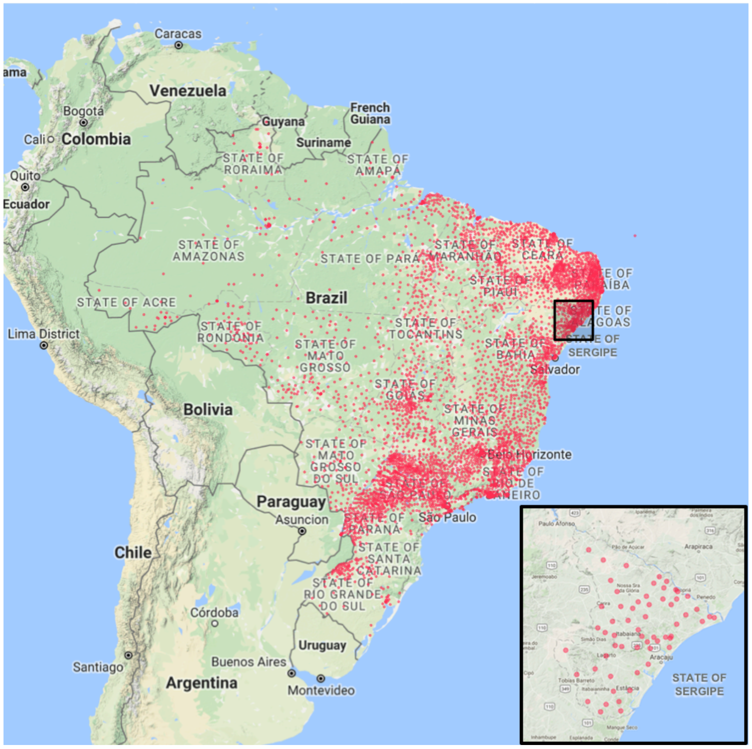

S6 Fig. Aedes aegypti mosquito map in Brazil. A map of *Aedes aegypti* mosquito occurrences in Brazil**.** Red dots indicate the location of each mosquito occurrence in 2013. In total there were 5057 mosquito occurrences in Brazil and 60 mosquito occurrences in the state of Sergipe.

Municipality Weighting

Aedes aegypti mosquitos have been shown to have higher reproductive rates as temperature increases (while below 32° C). And similar population increases have been demonstrated in regions with higher rainfall. Given these entomological features, we weight municipalities that have conditions suitable to large *Aedes aegypti* mosquito populations. We do this by randomly generating additional points in a 5km radius around municipalities in the top 5% most habitable municipalities based on these climate heuristics. The simplicial complex that we build through the Vietoris-Rips filtration then reflects this weighting since municipalities that are more habitable will have more 0D and 1D features.

Country-level Temperature

We obtained mean monthly temperatures in degrees Celsius from 98 weather stations which cover all states of Brazil in 2010 using FAOClim-Net, an agroclimatic database management system [3]. The data is available at http://geonetwork3.fao.org/climpag/. We take the average of these monthly temperatures to obtain a mean annual temperature for each state of Brazil in the year 2010. We use these temperatures as a proxy for the temperatures of each state of Brazil in recent years.

Municipality-level Temperature and Rainfall

Data on average monthly temperature between March to May in Brazil on a municipality level is available at http://www.ipeadata.gov.br.

Population Density
We obtained population data and geographic area (km) for each state of Brazil from the Instituto Brasileiro de Geografia e Estatistica (IBGE) [4]. We use the estimated resident populations in states of Brazil in the year 2014. To obtain an estimated population density for each state, we divide the estimated resident population data by the geographic area (km). The data can be downloaded at http://downloads.ibge.gov.br/downloads_estatisticas.htm

Zika Cases
The data on the number of Zika cases in each state of Brazil come from monthly reports published by Brazil's Ministry of Health [5]. Although the Ministry of Health publishes cases that are under investigation, we only used data on the confirmed cases. We used the latest cumulative data published weekly, which is from July 2, 2016. There are 27 states in Brazil, so we have 27 data points. The data can be obtained from the PDF available at: http://portalsaude.saude.gov.br/index.php/o-ministerio/principal/leia-mais-o-ministerio/197-secretaria-svs/20799-microcefalia

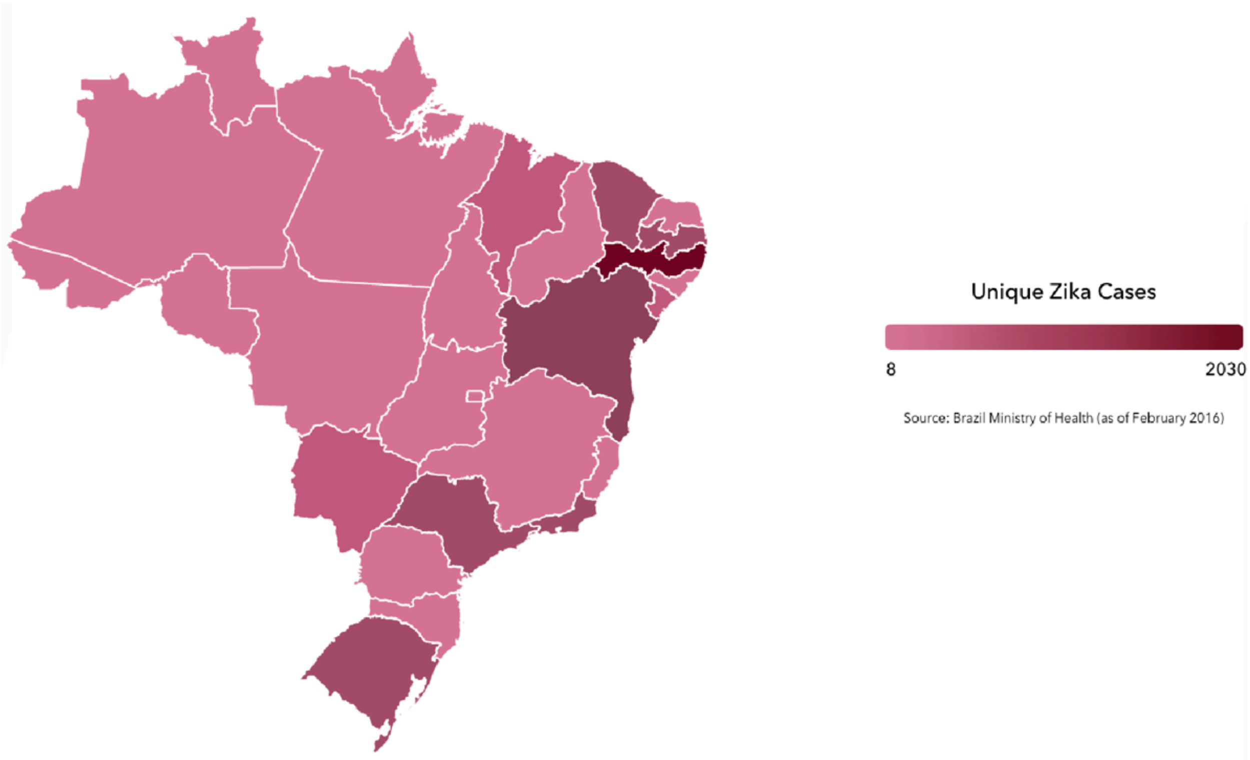

S7 Fig. Aedes aegypti heatmap in Brazil. A heatmap of confirmed Zika cases in Brazil.

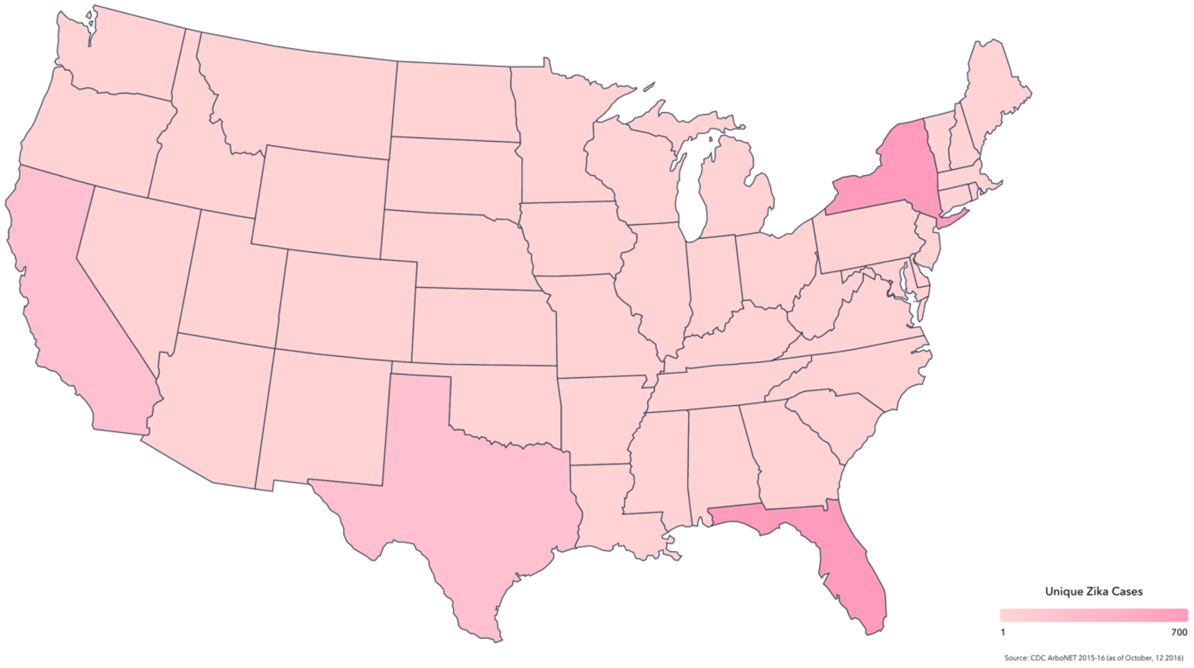

S8 Fig. Heatmap of confirmed Zika cases in United States. A heatmap depicting the unique confirmed Zika cases in the United States. When compared to S7, it is clear that there are far more confirmed cases in Brazil than in the United States. The maximum number of Zika cases in the United States is in Florida at 700. By comparison, the maximum number in Brazil is 2030, in Pernambuco.

References

1. Carlsson, Gunnar. "Topology and data". AMS Bulletin 46(2), 255–308 (2009).
2. Kraemer MUG, *et al.* The global compendium of Aedes aegypti and Ae. albopictus occurrence. Scientific Data 2 (7): 150035. http://dx.doi.org/10.1038/sdata.2015.35 (2015)
3. FAO. FAOClim-NET. x_203_mntmp_1480565212. Latest update: 12/31/2010. Accessed 10/23/2016. http://geonetwork3.fao.org/climpag/ (2010).
4. IBGE Estimates – Estimates of resident population in Brazil, federative units and municipalities. IBGE.gov.br (2014).
5. Ministry of Health. Brazil. http://portalsaude.saude.gov.br/index.php/o-ministerio/principal/leia-mais-o-ministerio/197-secretaria-svs/20799-microcefalia (2016)
